# The experiences of districts in implementing a national incentive programme to promote safe delivery in Nepal

**DOI:** 10.1186/1472-6963-9-97

**Published:** 2009-06-09

**Authors:** Timothy Powell-Jackson, Joanna Morrison, Suresh Tiwari, Basu Dev Neupane, Anthony M Costello

**Affiliations:** 1 Department of Public Health and Policy, London School of Hygiene and Tropical Medicine, Keppel Street, London, UK; 2 Centre for International Health and Development, Institute of Child Health, University College London, London, UK; 3 Support to Safe Motherhood Programme, Kathmandu, Nepal; 4 Independent Consultant, Kathmandu, Nepal

## Abstract

**Background:**

Nepal's Safe Delivery Incentive Programme (SDIP) was introduced nationwide in 2005 with the intention of increasing utilisation of professional care at childbirth. It provided cash to women giving birth in a health facility and an incentive to the health provider for each delivery attended, either at home or in the facility. We explored early implementation of the programme at the district-level to understand the factors that have contributed to its low uptake.

**Methods:**

We conducted in ten study districts a series of key informant interviews and focus group discussions with staff from health facilities and the district health office and other stakeholders involved in implementation. Manual content analysis was used to categorise data under emerging themes.

**Results:**

Problems at the central level imposed severe constraints on the ability of district-level actors to implement the programme. These included bureaucratic delays in the disbursement of funds, difficulties in communicating the policy, both to implementers and the wider public and the complexity of the programme's design. However, some district implementers were able to cope with these problems, providing reasons for why uptake of the programme varied considerably between districts. Actions appeared to be influenced by the pressure to meet local needs, as well individual perceptions and acceptance of the programme. The experience also sheds light on some of the adverse effects of the programme on the wider health system.

**Conclusion:**

The success of conditional cash transfer programmes in Latin America has led to a wave of enthusiasm for their adoption in other parts of the world. However, context matters and proponents of similar programmes in south Asia should give due attention to the challenges to implementation when capacity is weak and health services inadequate.

## Background

Programmes that provide monetary incentives conditional on households engaging in certain health seeking behaviours have been popular for the past decade, particularly in Latin America. Such programmes have been implemented at scale, and typically target poor families and children [1,2]. Conditional cash transfers can be an effective means to increase utilisation of preventive health services and, in some cases, improve health outcomes [3,4]. However, the literature also points to problems, including: the inefficiencies of paying some people to do what they would have done anyway [5]; the high administrative cost of programmes [6]; the potential for unintended effects [7]; and ethical concerns, such as those related to the promotion of permanent contraceptive methods [4].

Interest in using cash incentives to influence behaviour and promote the health of families has spread to south Asia. Progress in raising the low coverage of skilled birth attendance (ie. delivery attended by either a doctor, nurse or midwife) in the region has been slow [8] and it is against this backdrop of relative stagnation that Nepal, India and Bangladesh have adopted policies to provide financial incentives to women in an effort to encourage greater use of maternity services [9-11]. While these policies are not intended to act as a social safety net – they are more orientated towards behavioural change – they still share many of the same characteristics as conditional cash transfer programmes.

This paper reports the findings of a qualitative study describing implementation of Nepal's scheme to understand the factors that have hindered uptake of the programme. Nepal's Safe Delivery Incentives Programme (SDIP) (formerly known as the Maternity Incentives Scheme) was launched in 2005, with the aim of raising the coverage of skilled birth attendance. It marked a departure from past government policy that had tended to focus predominantly on service delivery without serious regard for demand side barriers. The establishment of the SDIP was a response to mounting evidence of the high cost faced by households trying to access care at childbirth [12] and the low coverage of skilled birth attendance, most recently estimated at 19 percent [13].

National implementation of the programme meant that there were few opportunities to pilot different implementation approaches and develop mechanisms to verify the eligibility of women, pay beneficiaries, and monitor the programme, elements deemed essential for implementation [1]. In countries like Nepal, with weak governance and limited capacity [14], the need for such systems is perhaps all the more vital. Concerns were also raised about the complexity of the programme and how this might affect implementation.

We aim to shed light on these issues and, more broadly, contribute to the small body of literature on health policy implementation in developing countries [15,16]. Our approach is broadly descriptive, seeking to identify what is happening in terms of the design, implementation, administration, and operation of the programme; whether it is expected; and the reasons behind why it is happening as it is [16,17]. We focused on the experiences of actors involved in the programme at the district level in order to give a bottom-up perspective of the implementation process. A description of the design of the programme and its implementation at the central level establishes the context in which district level actors were operating. An important part of the story, namely the formulation of the policy, has been described elsewhere [18].

### Safe Delivery Incentives Programme

The SDIP sought to change the behaviour of both families and health workers though a package of financial incentives (table 1) that included: i) a conditional cash transfer to women; ii) an incentive to the health provider for each delivery attended; and iii) free health care, in addition to the conditional cash transfer, for those women from the 25 least developed districts [11]. There have been a number of changes made to the design of the SDIP since our study. Here, we describe the benefits and eligibility criteria that were in place during the study period – ie. the first 18 months of the programme.

**Table 1:** Financial incentives offered by the SDIP and the eligibility criteria

Financial incentive	Eligibility criteria
1. Conditional cash transfer to women ▪ 500 NRS ($7.8) in plains districts ▪ 1,000 NRS ($15.6) in hill districts ▪ 1,500 NRS ($23.4) in mountain districts	Woman delivered in a public health facility and had no more than two living children or an obstetric complication (as diagnosed by the health provider)
2. Provider incentive ▪ 300 NRS ($4.7) for each delivery attended	Doctor, nurse, midwife, health assistant, auxiliary health worker or maternal and child health worker attended a delivery at the woman's home or in a public health facility
3. Free delivery care to women and facility reimbursed ▪ 1,000 NRS ($15.6) reimbursed to health facility	Woman comes from one of the 25 least developed districts and meets the eligibility criteria required for the conditional cash transfer

The SDIP offered cash to women giving birth in a public health facility. Money was to be paid by the health provider or accountant on discharge and the amount was set to reflect differences in accessibility to health facilities across the three main geographical regions of Nepal (table 1). In contrast to many performance-based payment schemes, the government chose to provide a universal conditional cash transfer rather than one targeted towards the poorest. This decision was driven by the desire to gain political popularity, as well as concerns about equity [18].

The SDIP intended to alleviate some of the transport costs of accessing care. The conditional cash transfer represented 30–50 percent of the mean transport cost incurred by a family seeking delivery care at a health facility [12]. Two groups of women were eligible to receive the money: i) women with up to two living children; and ii) women with any number of specified obstetric complications, irrespective of parity. The rationale for the first of these eligibility criteria was to avoid a risk of the SDIP increasing fertility. Clearly, the use of cash rather than a voucher system meant that money had to be transferred from the district health office to the health facility and, once there, securely kept. In many health centres with no accountant or bank account, this implied the handling of large sums of cash by health workers.

The SDIP also provided 300 NRS ($4.7) to health workers for each delivery attended. Surprisingly, perhaps, the provider incentive was given for deliveries attended both at the health facility and at home. Policymakers were anxious that the SDIP should not neglect home deliveries altogether, as it was unrealistic to expect the majority of women, even over the medium term, to deliver in health facilities given the current low coverage rate. However, this decision gave rise to a possible tension with the conditional cash transfer, on the one hand, incentivising institutional deliveries and the provider incentive, on the other hand, encouraging health workers to attend home deliveries. The policy specified the cadres of health worker eligible to receive the incentive, but was intentionally vague on whether or how the incentive should be shared amongst health workers, leaving the decision to the health facility management committee. Initially, auxiliary health workers were not eligible for the provider incentive, but after one year of widespread protests, the policy was changed to include them.

Free maternity care was available to women from the 25 least developed districts as an additional benefit to the conditional cash transfer. The policy stipulated that health facilities were to be given 1,000 NRS ($15.7) for each delivery provided free of charge, as a reimbursement to recover costs.

### Central level implementation

The central government issued guidelines to each district health office, zonal hospital and regional hospital on the how the SDIP should be designed and implemented [11]. In some respects (eg. sharing of the provider incentive), the guidelines gave actors at the district level discretion over implementation. On other matters (eg. the eligibility criteria), they represented a set of regulations that were intended to be strictly applied. In the absence of training, the guidelines were the primary means by which the policy was communicated to implementers at the district level.

There were lengthy delays in the transfer of funds from the central level (including the donor) to the districts. On average, districts received funds earmarked for the SDIP 283 days late in the first fiscal year, and 147 days late in the second fiscal year [19]. To initiate implementation of the SDIP as quickly as possible, the central level government encouraged districts to start paying beneficiaries using funds from other budget lines or borrow money from local partners. Wary of raising expectations amongst the public before ensuring funds were available to pay beneficiaries, the central level programme managers chose not to publicise the SDIP with a national media campaign. To be precise, the SDIP's launch was publicly announced; however, there was no national information campaign explaining the benefits of the programme and who could benefit

### Uptake of the programme

Available data suggest uptake of the programme in its first two years was, broadly speaking, low. The most reliable data, from a household survey carried out in six districts, indicate that 29 percent of eligible women received the conditional cash transfer at the time of childbirth and only 27 percent of women had any knowledge of the programme while they were pregnant [19]. There were found to be wide variations between districts, suggesting implementation has been more successful in some places than others. The proportion of eligible women receiving the cash ranged from 16 percent to 52 percent, while awareness during pregnancy varied from 22 percent to 40 percent. Over the same period, annual expenditure of the programme was $1.2 million with approximately 50 percent going to women recipients of the conditional cash transfer [20].

## Methods

### Study setting

Nepal is a geographically and ethnically diverse country that has suffered from political conflict and internal upheaval as the country moved from democracy to monarchy and now to an interim phase of political uncertainty [21]. Himalayan mountains border Tibet to the north, and India borders the flat plains to the south. Gross national income per capita is $340 [22] and while female literacy is improving, 45 percent of women remain illiterate [13]. Nepal has a maternal mortality rate of 281 per 100,000 live births and a neonatal mortality rate of 33 per 1,000 live births, and only 19 percent of women deliver with a skilled birth attendant [13].

### Study participants and data collection

The study was conducted in 10 districts of Nepal. We purposively sampled two mountain districts, four hill districts, and four plains districts to ensure that the variation in the SDIP's package of benefits across the three topographical regions was represented. In each district, we sampled five health institutions that were providing delivery care (one hospital, two primary health centres and two health posts). Health facilities were purposively selected to include two that were near to the district health office and two that were further away. In addition, two regional hospitals were sampled from other districts since our study districts contained no hospitals providing this level of care.

We aimed to conduct one key informant interview with the most senior level of health personnel available in each health facility, a key informant interview with the district health office and a focus group discussion in each district. Participants of each focus group discussion were a mix of health personnel, accountants, non-governmental organisation workers, and management committee members. In total, 55 key informant interviews and nine focus group discussions were completed (table 2). Trained Nepali researchers conducted discussions and kept log-books. TPJ and ST worked from the offices of Family Health Division in the Ministry of Health and Population, and made notes of their observations during this time. Verbal data were tape-recorded and transcribed. The study was approved by the ethics committee of the London School of Hygiene and Tropical Medicine and written consent was obtained from each respondent.

**Table 2:** Number of key informant interviews completed and analysed (in parentheses) by place

	Plains	Hill	Mountain	Total
District health office	3 (2)	3 (2)	1 (1)	7 (5)
Regional hospital	0 (0)	2 (2)	0 (0)	2 (0)
Zonal/district hospital	6 (3)	4 (3)	1 (1)	11 (7)
Primary health centre	8 (6)	7 (6)	3 (2)	18 (14)
Health post	7 (6)	6 (5)	4 (3)	17 (14)
				
Total	24 (17)	22 (18)	9 (7)	55 (42)

Note: In addition, there were nine focus group discussions completed and analysed (four in plains, three in hill and two in mountain districts)

### Analysis

Researchers who had collected and transcribed the data conducted manual content analysis [23] with two international researchers (TPJ, JM). Initially, 10 transcripts from different types of respondents and different topographical areas were categorised under headings from the topic guides. Categories were refined based on themes emerging from the data, and thereafter, transcripts were analysed according to those themes. We analysed all the focus group discussion data and a random sample of respondents in every district, ensuring coverage of stakeholders and methods (table 2). After analysis of 51 transcripts we felt there was saturation and a further 9 transcripts were read to check for recurring themes. Data were tabulated to compare responses across types of respondent and topography. Analysis was conducted in Nepali, and quotations were translated into English for publication. The extent to which observation data corroborated data from transcripts was discussed during analysis, and an observation report was written, describing the implementation of the programme.

## Results

### Variations in implementation

Information on examples of implementation were obtained from respondents in interviews and focus group discussions, allowing us to aggregate specific cases into categories of practices that could be mapped against the stated policy (table 3).

**Table 3:** Variations in implementation

Policy	Practice
*1. Conditional cash transfer to women*	
Women who deliver in a public health facility – hospital, primary health centre or health post – are entitled to a cash payment if they have no more than two living children or an obstetric complication. The cash is given at the time of discharge. If referred, the woman is to receive the cash from the referring institution.	▪ Cash given to women delivering at home ▪ Cash given to women delivering in sub-health posts ▪ Cash given to all women, and not limited to those with no more than two living children or an obstetric complication ▪ Reduced amount of cash given to women, because the health institution did not have sufficient funds and equally divided the money among the eligible women ▪ Cash amount deducted from the patient's bill in the absence of any cash to hand over to the woman ▪ Cash given on a first come first served basis or selectively due to insufficient funds ▪ Cash not given at time of discharge but at a later time ▪ Cash given to the relatives of women on proof of identity ▪ Cash given to women with abortion complications (not in the policy) ▪ Cash given to women by the referral hospital, rather than the referring institution ▪ No cash given
*2. Health provider incentive*	
Government health professionals – medical doctor, nurse, midwife, health assistant, auxiliary health worker and maternal and child health worker – are entitled to an incentive for every delivery they attend either at home or at a government health facility	▪ Incentive to health workers given only for attendance at those deliveries in which the woman has no more than two living children or an obstetric complication ▪ Incentive divided up among all staff involved in delivery care ▪ Incentive given to a single health professional involved in the delivery ▪ A share of the incentive taken by non health professionals – eg. the accountant ▪ Incentive given to health professionals for attending institutional deliveries only, not home deliveries ▪ Incentive claimed by health professionals for attending deliveries in private clinics ▪ No incentive given to health professionals
*3. Free delivery care to women*	
Women eligible for the cash incentive and resident in a low human development district are also entitled to free delivery care. The health institution is reimbursed 1,000 NRS for each case of free delivery care given	▪ Free delivery provided to all women, and not limited to those with no more than two living children or an obstetric complication ▪ Health institution not reimbursed for the free delivery

There were numerous practices in the implementation of the conditional cash transfer to the women. There were variations in the interpretation of the eligibility criteria and the administration of the cash. The former included cases where health facilities simply ignored the eligibility criteria altogether, making the cash available to all women delivering in a public health facility. More serious deviations from policy were apparent when an entire district, for example, gave cash to women delivering at home. Such practices might be expected to have the opposite effect to the SDIP's intended objective of increasing skilled birth attendance.

Administration of the cash varied in terms of: the amount of cash given; to whom the cash was given; and the time at which the cash was given. The practice of giving cash to husbands was a particular concern to some respondents who were worried that the money might be misspent. Most commonly, women were given no money at all or given it at a later date after being discharged from the health facility.

There were many instances of health facilities following their own rules in the payment of the health provider incentive. Practices varied according to who was entitled to receive the incentive, between whom the incentive was shared, and the place of delivery for which health workers could claim the incentive. Different practices regarding the sharing of the incentive between health staff were expected given that the policy guidelines allowed health facility management committees discretion on this issue. Some health facilities, however, took practices a step further, including non-health workers, such as the accountant, in the share of the incentives. The free delivery care element of the policy was usually implemented as intended. Sometimes, respondents reported that the health facility had not been reimbursed for the deliveries provided free of charge.

### Factors affecting implementation

#### Delays in disbursement, inadequate funds and their consequences

 Respondents at all levels of the health system expressed concern that women were not getting the incentive immediately after delivery. Often, money was not given to women in time because of delays in money reaching districts, inadequate funds or a combination of these factors. Delays occurred because of the late arrival of fund release letters from the central level, and the short-term absence of key district staff who were in the field or in training. In a few districts, health workers were also experiencing long delays before receiving their incentive, but more concern was expressed about the failure to pay women on time, particularly because the purpose of the cash was to meet immediate expenses of travel and subsistence.

"As the incentive is given for transportation expenses it should be given on time." (District hospital, Hill, Key informant interview)

"In my opinion, the incentive should be given to the woman immediately after she delivers...if she does not get the money in her hand she may not be able to borrow and then she will face problems." (District hospital, Hill, Key informant interview)

"We sometimes feel that this scheme should be stopped. Either the money should arrive on time otherwise it does not have any meaning." (District hospital, Hill, Key informant interview)

A diverse group of respondents also reported that funds had been inadequate to pay beneficiaries. The amount budgeted did not match requirements, or the amount released was less than the amount stated in the budget. There was frustration that the central level did not consult with the district level in the development of plans, even when the district had taken the initiative to plan how to spend the budget.

When money was not available to give to eligible women at the time of delivery, a number of issues arose. First, it was difficult to find or contact women in order to give them the money, particularly those in remote areas or those whose whereabouts were unknown. Outstanding debts accumulated and many women, at the time of this study, were yet to be paid:

"Since we have no money during the time of delivery we have to ask [women] to come later to collect the incentive. It will be difficult to find them later on, and many do not contact us. You see the problem?" (Health post, Hill, Key informant interview)

"Women come from 60 kilometres away for delivery. If they do not receive the money immediately after delivery, how many times can they come from so far, just for 500 rupees, and how many times can they call?" (District stakeholders, Plains, Focus group discussion)

Second, a failure to give the incentive created a perception that health institutions were withholding money, leading to friction and mistrust of health personnel:

"We started working with a tentative plan but as the number of deliveries increased, women who delivered in the beginning got the incentive and others were left out. About 40 to 50 women did not get the incentive. Those who did not get [the money] started fighting with us." (District stakeholders, Hill, Focus group discussion)

In one place, the programme was put on hold because there were inadequate funds, and staff were worried about the consequences of only giving cash to some women:

"If we distribute the outstanding incentive of last year from February onwards we need about five hundred thousand rupees but we have received only one hundred thousand. If we distribute this amount we are sure to be beaten by women." (District stakeholders, Mountain, Focus group discussion)

A number of ways to deal with the unpredictability of funds were described. These included: making payment on a first come first served basis; providing money out of one's own pocket; giving a smaller amount of money to share the cash across a larger number of women; and borrowing from other sources. Some district health offices used funds from other health programmes, whilst some health institutions borrowed from their own account. Some district officials, who were reluctant to borrow, worried that donor funds may not materialise, or felt it was risky to borrow without approval.

"If we spend money from the regular budget head funded by foreign donors we are questioned. If we spend even after receiving an authorisation letter we are questioned: 'why did we spend without receiving the letter of release?' Even those taking risks are trapped sometimes." (District health office, Plains, Key informant interview)

"I am reluctant to use money from the hospital committee fund because donor funds are highly unpredictable and we do not know when they will come." (District health office, Mountain, Key informant interview)

#### Confusion about the policy

Many respondents were unclear about aspects of the policy. They reported that there had been inadequate dissemination of information to the districts. The confusion created variations in implementation and affected the ability of health workers to disseminate information in communities, potentially hampering the effectiveness of the programme.

District officials and health workers were often confused about how to implement the programme, finding the official guidelines issued by central government unhelpful, confusing and lacking in detail. There were reports of late distribution of guidelines and insufficient copies, which restricted their ability to implement the policy:

"The centre was so miserly to send only one guideline. How do they think that this is sufficient to run [the programme] in such a big district?" (District stakeholders, Plains, Focus group discussion)

"Most importantly, the staff should be properly oriented before launching the programme." (Health post, Mountain, Key informant interview)

There was particular confusion about the eligibility of health workers to receive the incentive, and the process of reimbursement to health institutions for free delivery care. Respondents did not know the cadres of health worker and the place of delivery that ensured eligibility to receive the money. Data suggest that the conditional cash transfer to women was more clearly understood, but there were still some issues of confusion that led to deviations from the stated policy. Sometimes cash was distributed to women at ineligible health facilities and women were not paid when they were referred:

"I am not sure about getting the incentive when referring the women to another facility after I attended her first. And I am also not sure from which facility the women will get the incentive..." (Primary health centre, Plains, Key informant interviews)

"We were all confused about how to distribute the incentive and we were not sure whom to give, [and] how much to give" (District stakeholders, Mountain, Focus group discussion)

Districts disseminated information to the community using various means, such as FM radio or through female community health volunteers. However, district stakeholders felt communication was hampered by a lack of guidance on how to promote the policy, and the absence of any budget allocation. Moreover, some respondents felt it difficult to disseminate information about a programme that they themselves did not fully understand. Disseminating information about the programme was particularly challenging in sparsely populated areas where households were scattered. Respondents felt that poorer women who tended to live in more remote areas were disadvantaged because they were less likely to know about the programme. Women in more remote areas who had not received the cash at delivery were also disadvantaged as they were less likely to find out that the budget had been released:

"Only advantaged women received the incentive as they are better informed and are able to visit the health facilities. The poor ones were not informed properly and did not receive the incentive." (District stakeholders, Plains, Focus group discussion)

"I am sure people in town know about the scheme but I am not sure about in the villages" (District stakeholders, Plains, Focus group discussion)

#### Misuse and monitoring

Respondents in several districts were concerned about misuse of SDIP funds. Different types of misuse were described, including: false claims by health workers for deliveries that never took place; claims by health workers for assisting deliveries in private clinics; claims for money by women with more than two children; and skimming of money by staff at the district level. Occasionally reports of false claims were followed up, but the lack of a budget provision hindered verification of parity or if a birth actually took place.

" [Health workers] are claiming incentives for deliveries that they have attended in the local market place, and registering them as institutional deliveries. And there is no mechanism to check." (District stakeholders, Plains, Focus group discussion)

"You know, sometimes the district level people keep up to fifty percent of the incentive. I heard they make false claims too." (Health post, Hill, Key informant interview)

In some cases, it was not clear whether the lack of clarity in the guidelines caused the (unintentional) misuse of funds, or if they were wilfully misused. Either way, respondents felt that there were opportunities for misappropriation of funds by virtue of the programme's design:

"When cash comes, there are possibilities for misuse, this is the only problem." (District health office, Hill, Key informant interview)

In almost all districts, respondents were unclear about what was expected of them in terms of monitoring the programme. There was a lack of guidance on how to monitor and there was no separate budget or time allocation to carry out these activities. Many felt that monitoring the distribution of financial incentives was important, but guidance had been insufficient.

"So far we have not monitored the programme and no one has raised any question about this. However, this now stands as a big issue." (District hospital, Hill, Key informant interview)

"A separate budget for supervision and monitoring of this scheme should be made available. The integrated budget for monitoring is just not enough." (District stakeholders, Hill, Focus group discussion)

No monitoring system had been established in any of the districts we sampled. Some respondents felt that they had not been given time to prepare recording and reporting tools, and hasty implementation had resulted in an inability to plan or set up a monitoring system. Reporting appeared to be on an ad hoc basis, provided 'on demand' to representatives of non-governmental organisations and central level officials, or in meetings where the programme was discussed. In some places respondents felt that health workers were not expected to monitor the scheme, and this was the responsibility of those at the central level or non-government organisations.

#### Acceptance of the policy

Most respondents welcomed the idea of giving cash to women delivering in a health facility and felt it was both legitimate and helpful. However, they had specific concerns with the eligibility criteria, questioning the logic of only giving the cash to women with two or fewer children. Respondents felt that this indirect discrimination, particularly against poorer women, opposed the overall aim of the programme – to increase institutional deliveries and reduce mortality:

"The poorest of the poor are excluded from the incentive because the poor are the ones who have more than two children. So there should not be any parity condition" (District health office, Hill, Key informant interview)

"Women having more babies are subject to higher risks and they are deprived of the incentive." (District stakeholders, Plains, Focus group discussion)

"If safe motherhood is women's right, then what about the rights of women having more than two children?" (District stakeholders, Plains, Focus group discussion)

Health workers sometimes found themselves in difficult situations, either being unable to provide the cash to poorer women with greater need because they had more than two children, or purposively ignoring the parity restriction:

"Women with 4 or 5 babies must also have been paid the incentive, because I have paid the incentive to women with 3 babies" (District stakeholders, Plains, Focus group discussion)

"It is difficult if rich people get money and poor people do not get money...for example, a rich woman came to the health institution for delivery and got the incentive. A poor woman found out about the incentive from that person but we could not give her the incentive because she had more than two babies...(this) makes me feel uneasy." (Primary health centre, Hill, Key informant interview)

Other respondents were concerned about the practicalities of reliably verifying the parity (and therefore eligibility) of women and methods of verification were ineffective and sometimes restrictive:

"How can a woman go to the Village Development Committee and get a certificate (of parity) before going to the health institution for delivery? Currently there is no local political representative, how can it be practical?" (District hospital, Hill, Key informant interview)

"The main barrier is to find out the number of babies, even a mother with 3 or 4 babies claims that they have just 2 babies, and we are not trained to find out the parity (of the woman)." (District hospital, Hill, Key informant interview)

There was widespread discontent with the health provider incentive, even amongst those who benefit directly such midwives. It strained relations between health staff, particularly when some felt the distribution of money was unjust or higher qualified staff were ineligible to receive the incentive:

"Distribution of incentives to health workers creates tensions especially in cases of home delivery. Often the home delivery is attended by one health worker but others who do not attend also the want the money to be equally distributed" (District stakeholders, Plains, Focus group discussion)

"The guidelines are discriminatory by giving money to maternal and child health workers and not auxiliary health workers who are in charge. This issue is highly controversial in my district." (Health post, Plains, Key informant interview)

In one large hospital, managers wanted to avoid any conflict between staff and therefore no incentive was provided to health workers. Instead, the money was used to make up the deficit in payments to women.

Respondents felt that effective implementation of the programme required improvements in the availability and quality of services. This typically manifested itself in a call for greater investment in drugs, equipment and training of skilled birth attendants:

"It does not help just to distribute money to women. The skills of all health workers should be improved by providing proper training." (District stakeholders, Plains, Focus group discussion)

Many respondents suggested that funds should be used to provide free delivery care, instead of allocation to the SIDP, and some felt that the programme should be needs based, targeting poorer women:

"It is not necessary to provide incentives to all women. It should only be provided to poor women, otherwise it is not worthwhile and it is a loss of money. On the contrary, rich women should be charged...because they can afford it." (Regional hospital, Hill, Key informant interivew)

"It would be even better if the amount of incentive could be given to the hospital to make the delivery service completely free." (District hospital, Hill, Key informant interview)

## Discussion

The paper has described the experiences of district level actors in implementing the SDIP and illustrates a number of challenges they faced. In the discussion, we expand on the most important issues raised by respondents with reference to the wider literature on the implementation of health policy in developing countries.

The study had a number of limitations. First, while it was always our intention to follow a descriptive approach to studying implementation [16], methods grounded in health policy analysis theory may have been able to provide deeper insights by stating a set of hypotheses to test empirically [24]. There are analytical frameworks, for example, that integrate "politics, process and power into the study of health policies" [15]. Second, we focused on early implementation only, motivated by the need to provide timely findings in the evaluation of an ongoing programme. We recognise this provides an incomplete picture, a snapshot of implementation over what is, at a minimum, a five-year programme. Third, all data collection was completed before the analysis of data was undertaken. A more iterative process of data collection and analysis would have improved the topic guides and experience of the interviewers.

Despite these limitations, the study was able to provide a detailed account of the SDIP's early implementation. We should not perhaps be surprised that early implementation faced several difficulties. It was a national programme which was introduced rapidly without time set aside for piloting or detailed planning at district level. The large variation in practices suggests district managers and health workers were able to exercise considerable discretion in the implementation of the programme and it is largely their actions that have determined how the policy took shape on the ground. At the same time, a number of factors at the central level imposed considerable constraints on the ability of actors in each of the districts to implement the SDIP. Bureaucratic delays in the disbursement of funds, difficulties in communicating the policy, both to implementers and the wider public and the design of the programme were cited by respondents as important. It is these factors that provide the most convincing explanation of why uptake of the programme was low. Poor financial management, in particular, has been a common theme emerging from the recent literature on implementation of maternal health financing policies in low-income countries [9,25,26].

Political expediency to ensure the policy was adopted quickly [18] may have meant there was inadequate preparation in the planning of resources and development of certain mechanisms. Respondents frequently spoke about the inadequacy of funds and acknowledged that the means to verify the eligibility of women and monitor the programme were lacking. Moreover, efforts by the central level to retain substantial control of the implementation process – by using an earmarked budget, providing prescriptive (yet unclear) guidance on the policy, and offering few opportunities for feedback – may have exacerbated problems and contributed further to the programme's low uptake.

In this context, the actions of district level actors may be seen as coping strategies to overcome (or avoid) the problems imposed on them from above and, in turn, they may explain the reasons for the variation in uptake of the programme between districts. Those who attempted to deal with challenges were often driven by pressure to meet local needs. Inaction, however, was equally common and it is important to understand the motivations that influenced the two sets of actors.

The most important coping strategy, given the bureaucratic delays at the central level, may have been the decision by some district managers to direct funds from other budget lines for the purposes of the SDIP. This practice presumably allowed implementation earlier than would otherwise have been possible, but its impact on the financing of other district health programmes is, from the data, unclear. Prior perceptions about the unpredictability of donor funding influenced the decision of some managers to refrain from using other budget lines and delay implementation. It is also possible that this strict adherence to government regulations may have represented a reaction – particularly among those who had taken the time to develop careful plans for how to allocate the SDIP budget between health facilities – against the continued pressure from the central government to use funds from other budget headings.

The fear of having to deal with angry families demanding their money appeared to be a motivating factor behind a number of practices to manage the problem of unpredictable funding to health facilities. Health workers tried to appease mothers by providing money out of their own pocket, deducting an amount equivalent to the conditional cash transfer from the hospital bill or sharing what money they had among all those waiting. Another motivating factor may have been the wide support for paying mothers – also found elsewhere [1,9] – which resonated with the view that mothers tend to put funds to better use than men. Some simply sidestepped the problem altogether, putting the programme on hold so that no women could claim the cash.

With respect to raising awareness, some districts undertook local initiatives to communicate information about the programme using, for example, FM radio. It suggests that other districts could have done more to promote the programme, but with widespread confusion among implementers they themselves may have lacked the confidence to take the initiative themselves.

It appears that the complexity of the programme – with the different eligibility criteria for different incentives – was itself a major barrier to implementation, exacerbated further by the confusing guidelines. There is no denying that such programmes are inherently complex; one only needs to looks at Brazil's conditional cash transfer programme, *Bolsa Familia *[27]. However, an alternative approach to implementation may have promoted more innovation and creativity at the district level with the emphasis on reaching policy objectives, rather than specifying the policy in such detail, which probably made people uncertain whether or not they could adapt the rules when faced with problems.

Central to this question of why some district implementers took actions to deal with problems and others did not may be the widespread knowledge that the SDIP was donor-funded. Interestingly, one of the reasons donors provide funds directly to the recipient government's treasury is to promote national ownership of policy. In practice, however, there appeared to be little ownership of the policy at the district level. The SDIP was perceived by many as a 'programme', whose activities were regarded as additional to the routine of government. Respondents maintained that monitoring, supervision and communication of the programme could not be carried out using available resources and the fact that there was no budget provision or explicit time allocation for these tasks reinforced the idea that they should be done by external monitors from the central level or NGOs. Other studies have found that such perceptions can be lead to inaction and a general reluctance to carry out tasks demanded of the central level [28-30].

Several issues arose that may have had serious implications for the wider public health system, highlighting the potential of the SDIP to have (unforeseen) adverse effects. The perception that health providers may have held money rather than paying women can only have eroded further the community's trust in the public health system, thereby reducing demand for all health services, not just delivery care. Equally serious, but from the perspective of service provision, was the tension between staff caused by the provider incentive. Any conflict between staff is likely to have consequences for all health services provided by the health facility. In this regard, the central level could have devoted more time to explaining the rationale for the provider incentive (ie. why only some cadres were eligible for the incentive) in order to build legitimacy of this element of the SDIP.

## Conclusion

Changes made to the SDIP since the study suggest development and implementation of the policy has been a dynamic process. In late 2007, the Government of Nepal made a number of changes, reflecting some of the most important concerns of respondents in this study. The eligibility criteria to receive the conditional cash transfer to women were removed, allowing all women to benefit and simplifying the programme enormously. The programme was expanded to include not-for-profit hospitals in order to address concerns about the availability of obstetric services. A national information campaign and district level training of staff were carried out to improve awareness and understanding of the programme. The orientation, in particular, provided a forum for district level staff to voice concerns and have greater exposure to information about the programme. Finally, procedures to manage and disburse funds at the central level were streamlined to reduce delays. These changes are welcome and they were most likely responsible for substantial increases in the uptake of the programme in its third year [19]. More recently, the government announced further changes, abolishing user fees for delivery care at all public health facilities while continuing to provide the SDIP's conditional cash transfer to women. Given the problems with early implementation and the adjustments made by policymakers, it will be important to evaluate the impact and implementation of the SDIP over the first five years.

The success of conditional cash transfer programmes in Latin America has led to a wave of enthusiasm for their adoption in other parts of the world. However, context matters and proponents of similar programmes in south Asia should give due attention to the challenges to implementation when capacity is weak and health services inadequate. There is a need for both careful planning in their roll out and closer engagement with district level actors, who exercise considerable influence in the implementation of policy.

## Declaration of competing interests

The authors declare that they have no competing interests.

## Authors' contributions

All authors contributed to the overall design of the study. TPJ, BDN and ST managed the data collection process. Data analysis was done by JM, BDN, ST and TPJ, in addition to a team of researchers involved in the data collection and trained by JM in qualitative data analysis methods. All authors drafted and approved the final manuscript.

## Pre-publication history

The pre-publication history for this paper can be accessed here:


